# Dynamic lactate indices as predictors of outcome in critically ill patients

**DOI:** 10.1186/cc10497

**Published:** 2011-10-20

**Authors:** Alistair Nichol, Michael Bailey, Moritoki Egi, Ville Pettila, Craig French, Edward Stachowski, Michael C Reade, David James Cooper, Rinaldo Bellomo

**Affiliations:** 1 Australian and New Zealand Intensive Care - Research Centre, School of Public Health and Preventive Medicine, Monash University, Commercial Road, Melbourne, VIC, Australia; 2 Department of Anaesthesiology and Resuscitology, Okayama University Hospital, 2-5-1 Shikata-cho, Okayama, Japan; 3 Department of Intensive Care, The Alfred Hospital, Commercial Road, Melbourne, VIC, Australia; 4 Department of Intensive Care, The Austin Hospital, Heidelberg Road, Melbourne, VIC, Australia; 5 Department of Intensive Care, The Western Hospital, Gordon Street, Melbourne, VIC, Australia; 6 Department of Intensive Care, Westmead Hospital, Darcy Road, Sydney, NSW, Australia; 7 Faculty of Medicine, University of Melbourne, Swanston Street, Melbourne, VIC, Australia

**Keywords:** lactate, hyperlactaemia, dynamic, intensive care unit, critical illness, mortality

## Abstract

**Introduction:**

Dynamic changes in lactate concentrations in the critically ill may predict patient outcome more accurately than static indices. We aimed to compare the predictive value of dynamic indices of lactatemia in the first 24 hours of intensive care unit (ICU) admission with the value of more commonly used static indices.

**Methods:**

This was a retrospective observational study of a prospectively obtained intensive care database of 5,041 consecutive critically ill patients from four Australian university hospitals. We assessed the relationship between dynamic lactate values collected in the first 24 hours of ICU admission and both ICU and hospital mortality.

**Results:**

We obtained 36,673 lactate measurements in 5,041 patients in the first 24 hours of ICU admission. Both the time weighted average lactate (LAC_TW24_) and the change in lactate (LAC_Δ24_) over the first 24 hours were independently predictive of hospital mortality with both relationships appearing to be linear in nature. For every one unit increase in LAC_TW24 _and LAC_Δ24 _the risk of hospital death increased by 37% (OR 1.37, 1.29 to 1.45; *P *< 0.0001) and by 15% (OR 1.15, 1.10 to 1.20; *P *< 0.0001) respectively. Such dynamic indices, when combined with Acute Physiology and Chronic Health Evaluation II (APACHE II) scores, improved overall outcome prediction (*P *< 0.0001) achieving almost 90% accuracy. When all lactate measures in the first 24 hours were considered, the combination of LAC_TW24 _and LAC_Δ24 _significantly outperformed (*P *< 0.0001) static indices of lactate concentration, such as admission lactate, maximum lactate and minimum lactate.

**Conclusions:**

In the first 24 hours following ICU admission, dynamic indices of hyperlactatemia have significant independent predictive value, improve the performance of illness severity score-based outcome predictions and are superior to simple static indices of lactate concentration.

## Introduction

In the critically ill, a higher admission blood lactate concentration is associated with a higher risk of death [1-8]. We recently reported that even within the current 'normal range' (< 2.00 mmol.L^-1^) a higher admission blood lactate concentration is associated with significantly increased hospital mortality [4], a finding which suggests that even the subtle perturbations of lactate homeostasis may be important.

An elevated blood lactate concentration (a 'static' index) at any time point must be due to an increase in its production, a decrease in its clearance, or both. Likewise, an increasing blood lactate concentration (a 'dynamic' index) must be due to increasing production, decreasing clearance, or both simultaneously [9-11]. Static derangements in lactate homeostasis during ICU stay have become established as clinically useful markers of increased risk of hospital and ICU mortality [1,3,4,12]. However, dynamic indices of lactate homeostasis, which describe not only magnitude but also duration and trend over time, may be even more useful in predicting outcome. In support of this hypothesis, a number of small single centre observational studies, principally in patients with severe sepsis and septic shock, have suggested that early changes in blood lactate concentration may be useful in identifying those at high risk of death [5,6,13-16]. Furthermore, one interventional study (*n *= 348) has suggested that interventions aimed at targeting a dynamic reduction in lactate (20% per two hours for the first eight hours) in the critically ill with an abnormal admission lactate level may be associated with reduced organ failure and increased survival [17]. However, the association between dynamic changes in blood lactate concentration during the first 24 hours of ICU admission and mortality has not yet been investigated in a very large heterogeneous cohort of critically ill patients. Furthermore, to our knowledge no study has compared the ability of dynamic compared to static indices of lactate homeostasis to predict mortality in the critically ill.

To study this association, we examined the relationship between six indices of lactate homeostasis in the first 24 hours of ICU admission and both hospital and ICU mortality. Three indices were static: i) admission lactate (LAC_ADM_), ii) maximum lactate (LAC_MAX24_), iii) minimum lactate (LAC_MIN24_), and three were dynamic: iv) time weighted lactate (LAC_TW24_), v) absolute change in (delta) lactate (LAC_Δ24_) and vi) percentage change in lactate (LAC_%Δ24_).

## Materials and methods

The data collection and data analysis for this study are part of ongoing de-identified data auditing processes across the participating hospitals, which have all waived the need for informed consent. The Austin Hospital Ethics Committee approved studies related to this database.

### Study population and data sources

The study population and data sources used in this study are similar to those previously reported by us, in a previous manuscript describing lactate homeostasis in the critically ill [4]. In brief, this four-centre retrospective investigation of a prospectively gathered intensive care database enrolled patients from January 2000 to October 2004. However, in this study a minimum of two lactate values collected over the first 24 hours were necessary for inclusion into the study, with the latter criteria needed to produce both a time weighted lactate (LAC_TW24_) and a change in lactate (LAC_Δ24_) over the first 24 hours.

The blood lactate concentration data used for this study were stored and retrieved electronically. We obtained age, sex, use of mechanical ventilation, reason for ICU admission (surgical and non-surgical, further classified as trauma, cardiac/vascular, gastrointestinal tract, neurological and thoracic/respiratory), and Acute Physiology and Chronic Health Evaluation (APACHE) II score [18] from the electronic data repositories of each ICU, using data prospectively collected as part of the Australian and New Zealand Intensive Care Society-Centre for Outcome and Resources Evaluation (ANZICS-CORE) quality assurance program [19].

The timing of lactate measurement (Rapilab, Bayer Australia, Sydney, NSW, Australia) was at the discretion of the managing critical care team. Laboratories in the participating hospitals comply with standards of the National Association of Testing Authorities [20] and the Royal College of Pathologists of Australia [21].

### Statistical analysis

**Figure 1:**
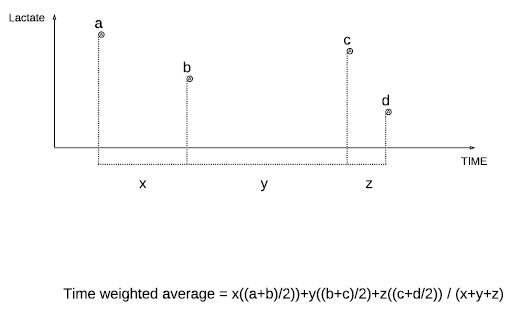
**Diagram describing the calculation of time weighted lactate (Lac_TW_)**.

To avoid the potential effect of surveillance bias due to the increased blood lactate monitoring in more severely ill patients, we calculated the time-weighted lactate concentration (LAC_TW24_). In brief, time weighted average lactate (Lac_TW_) is an index of lactate homeostasis that is proportional to the amount of time spent at each concentration in relation to the total period of time observed. It is determined by summing the mean value between consecutive time points multiplied by the period of time between consecutive time points and then dividing by the total time (see Figure 1). This method was modified from and used in accordance with an approach previously used by Finney *et al*. to describe hyperglycaemia [22].

The change in lactate over 24 hours (LAC_Δ24_) was calculated by regressing lactate against time for each individual patient, with the regression slope representing the projected change over a 24-hour period. To avoid undue influence from extreme outliers, the maximal and minimal slope values were capped in accordance with Tukey [23]. Values that were found to be more than three times the interquartile range to the left of the 25^th ^percentile or to the right of the 75^th ^percentile were considered to be extreme outliers. This resulted in a maximum increase or decrease in slope over the first 24 hours of 5 mmol.L^-1^. LAC_%Δ24 _describes the change in lactate over a 24-hour period as the percentage change from the admission blood lactate concentration.

The primary outcomes for analysis were hospital and ICU mortality. We performed univariate analysis for comparison between groups according to hospital mortality status using chi-square test for proportions, Student *t*-tests for normally distributed outcomes and, otherwise, used Wilcoxon rank sum tests. We performed multivariate analysis with all available predictors of hospital mortality included in the models (gender, age, APACHE II, mechanical ventilation, surgical admission and diagnosis type). To account for changes in practice over the four-year period of the study, the patient admission date was included along with the specific hospital. To further account for surveillance bias, the number of lactate measurements collected over the first 24 hours was also included. After forcing all of the previous described variables into the multivariate model, both stepwise and backwards elimination procedures were used to determine which lactate indices could independently aid in the prediction of mortality. Six lactate indices specifically relating to the first 24 hours of ICU admission were considered: Three indices were static: i) admission lactate (LAC_ADM_), ii) maximum lactate (LAC_MAX24_), iii) minimum lactate (LAC_MIN24_), and three were dynamic: iv) time weighted lactate (LAC_TW24_), v) absolute change in (delta) lactate (LAC_Δ24_) and vi) percentage change in lactate (LAC_%Δ24_).

To determine if the relationship between lactate and mortality was consistent across patient subgroups and study hospitals, we examined interactions between measures of lactate and other variables in the model. To confirm that the potential relationships between lactate and mortality were linear in nature, measures of lactate were divided into quintiles and analysed as categorical variables, with quintiles chosen to provide a minimum of 1,000 patients per category. Goodness of fit was determined using the Hosmer and Lemeshow statistic. All analyses were performed using SAS version 9.2 (SAS Institute Inc., Cary, NC, USA). A two-sided *P*-value of 0.05 was considered to be statistically significant.

Having established which lactate variables had the strongest relationships with mortality, to then determine the true predictive capacity of these lactate variables, the data were randomly divided into two groups, with 50% of the data used as a derivation sample and the remaining 50% of the data used as a validation sample. Univariate models and multivariate models (with and without the inclusion of lactate) were constructed from the derivation sample for both hospital and ICU mortality. The prediction equations were then applied to the holdout sample and the improvement in the Area under the Receiver Operating Characteristic Curve (AUC-ROC) was recorded.

## Results

We studied a heterogeneous cohort of 7,155 critically ill patients of whom 5,041 had at least two measurements over the first 24 hours (median 7; range 2 to 34) and were included in the analysis. All indices of blood lactate concentration over the first 24 hours (LAC_ADM_, LAC_TW24_, LAC_MAX24_, LAC_MIN24_), were significantly higher in hospital non-survivors compared to survivors (Table 1). The median decline in lactate over the first 24 hours (LAC_Δ24_) and the median percentage decline in lactate (LAC_%Δ24_) were significantly lower in non-survivors compared to survivors (Table 1).

**Table 1:** Comparison of hospital survivors vs.nonsurvivors

Variable	Non survivors (*n *= 974)	Survivors (*n *= 4,067)	*P*-value
Male sex	59% (574)	62% (2,510)	0.11
APACHE II score	24.7 (8.0)	15.3 (6.7)	< 0.0001
Age (yr)	65.7 (16.0)	59.7 (18.9)	< 0.0001
Mechanical ventilation rate	84% (817)	60% (2443)	< 0.0001
Surgical patients	32% (307)	51% (2087)	< 0.0001
Diagnosis at admission			
Cardiac and vascular	27% (265)	24% (979)	0.04
Thoracic and respiratory	20% (191)	18% (719)	0.16
Trauma	3% (27)	8% (343)	< 0.0001
Neurological	18% (175)	12% (485)	< 0.0001
Gastrointestinal tract diseases	13% (128)	21% (870)	< 0.0001
Other	19% (188)	16% (671)	0.04
Hospital stay (days)	10 (4 to 25)	15 (8 to 30)	< 0.0001
ICU stay (days)	3.0 (2.0 to 8.0)	3.0 (2.0 to 5.2)	< 0.0001
Number of measurements	8 (6 to 10)	7 (5 to 9)	< 0.0001
LAC_TW24 _(mmom.L^-1^)	2.20 (1.41 to 3.66)	1.41 (1.02 to 1.97)	< 0.0001
LAC_Δ24 _(mmom.L^-1^)	-0.21 (-1.66 to 0.73)	-0.30 (-1.18 to 0.24)	0.009
LAC_%Δ24_	-13% (-49% to 42%)	-22% (-55% to 21%)	< 0.0001
LAC_ADM _(mmom.L^-1^)	2.36 (1.45 to 4.29)	1.6 (1.08 to 2.49)	< 0.0001
LAC_MIN24 _(mmom.L^-1^)	1.34 (0.92 to 2.16)	1.00 (0.74 to 1.31)	< 0.0001
LAC_MAX24 _(mmom.L^-1^)	3.44 (1.99 to 6.20)	2.02 (1.37 to 3.20)	< 0.0001

APACHE II, Acute Physiology and Chronic Health Evaluation II; LAC_ADM_, admission lactate; LAC_TW24_, time weighted lactate; LAC_MAX24_, maximum lactate; LAC_MIN24_, minimum lactate; LAC_Δ24_, delta lactate; LAC_%Δ24_, percentage change in lactatein the first 24 hours

### Univariate analysis

We assessed the ability of both static (LAC_MAX24 _and LAC_MIN24_) and dynamic (LAC_TW_, LAC_Δ24 _and LAC_Δ24%_) indices of lactate homeostasis in the first 24 hours to predict hospital and ICU mortality benchmarked against LAC_ADM_, the most commonly used static measure (Table 2). The combination of LAC_TW _and LAC_Δ24_, which assesses duration, magnitude and trend of lactate derangement, resulted in the most significant increase in ability to predict hospital death (Table 2).

**Table 2:** Change in RAW (AUC-ROC) for indices of lactate homoeostasis in the first 24 hours and mortality

_NAME_	Hosp AUC-RAW	*P*-value*	ICU AUC-RAW	*P*-value*
LAC_ADM_	0.648 ± 0.014		0.700 ± 0.017	
LAC_Δ24%_	0.558 ± 0.016	0.0002	0.572 ± 0.022	< 0.0001
LAC_Δ24_	0.534 ± 0.016	< 0.0001	0.542 ± 0.022	< 0.0001
LAC_MAX24_	0.694 ± 0.014	< 0.0001	0.769 ± 0.016	< 0.0001
LAC_TW24_	0.705 ± 0.014	< 0.0001	0.769 ± 0.016	< 0.0001
LAC_MIN24_	0.682 ± 0.014	0.002	0.723 ± 0.018	0.15
				
LAC_TW _and LAC_Δ24_	0.710 ± 0.01	< 0.0001	0.763 ± 0.01	< 0.0001

*compared to LAC_ADM_

AUC-RAW, Unadjusted Area Under Curve; LAC_ADM_, admission lactate; LAC_TW24_, time weighted lactate; LAC_MAX24_, maximum lactate; LAC_MIN24_, minimum lactate; LAC_Δ24_, delta lactate-change in lactate in the first 24 hours; LAC_%Δ24_, percentage delta lactate-percentage change in lactate in the first 24 hours Hosp, hospital mortality; ICU intensive care unit mortality

### Multivariate analysis

**Figure 2:**
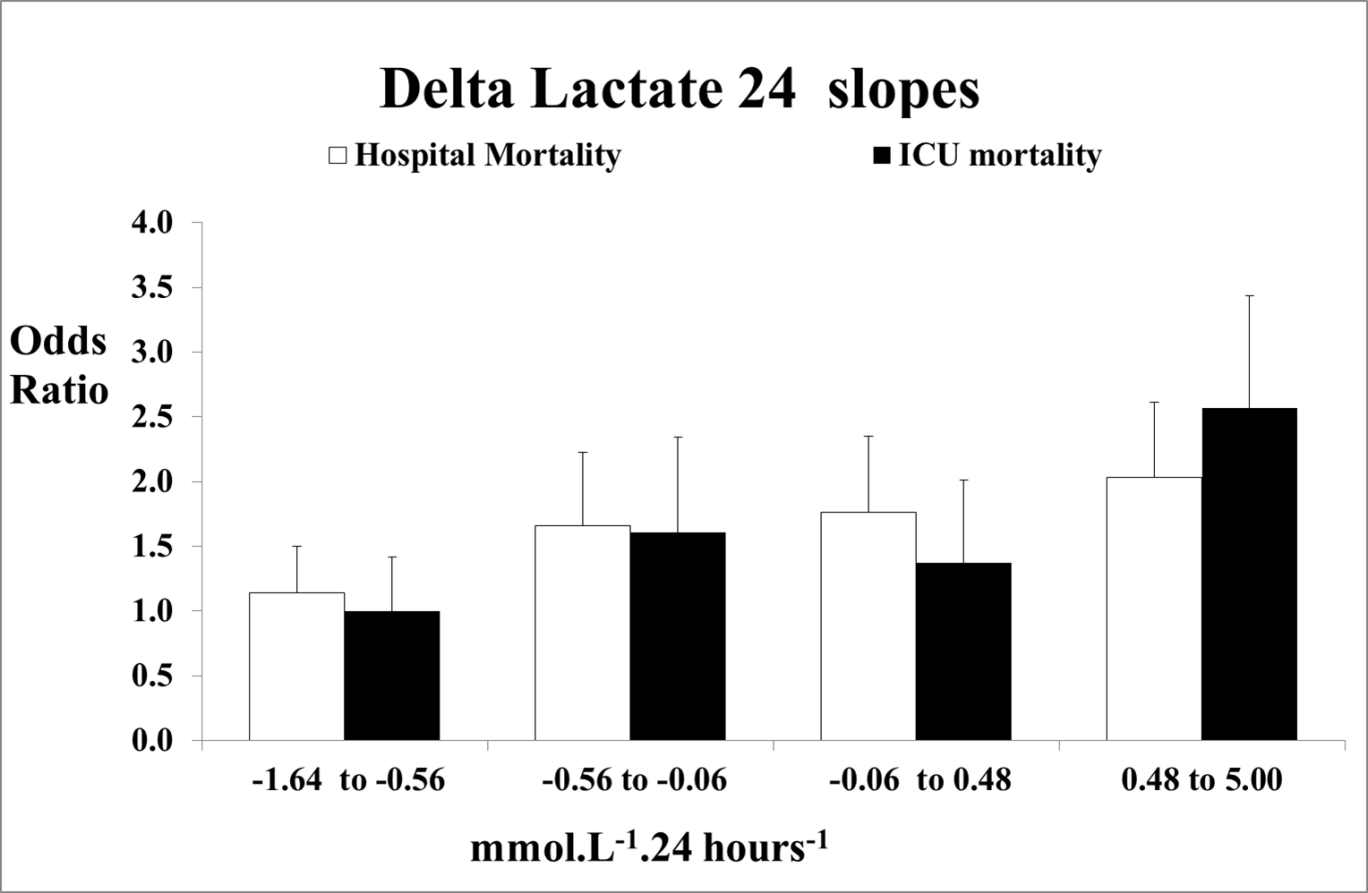
Adjusted* odds ratios for Lac_Δ24 _referenced against the lowest quintile. * adjusted for APACHE II, diagnosis, age, gender, ventilation, surgery, admission date, hospital number of measurements. 95% CI, 95% confidence interval; APACHE II, Acute Physiology and Chronic Health Evaluation II; LAC_Δ24_, delta lactate, change in lactate in the first 24 hours_; _Lac_Δ24 _change in lactate over the first 24 hours.

After adjusting for established predictors of mortality, the lactate variables that were independently the most predictive of hospital and ICU mortality were LAC_TW24 _and LAC_Δ24 _(Table 3). For every one unit increase in LAC_Δ24 _the risk of death increased by 15% (OR 1.15 (1.10 to 1.20) *P *< 0.0001) for hospital mortality and 18% for ICU mortality (OR 1.18 (1.13 to 1.24) *P *< 0.0001). When LAC_Δ24 _was divided into quintiles the relationship between LAC_Δ24 _and mortality also appeared to be well approximated by linearity (Figure 2). When the risk of death was calculated based on the slope of the LAC_Δ24_, an increasing blood lactate concentration (slope greater than 0 mmom.L^-1^.24 hours^-1^) was associated with a greater increase in risk of hospital and ICU death (OR 1.46 (1.23 to 1.74) *P *< 0.0001 and OR 1.71 (1.37 to 2.13) *P *< 0.0001, respectively). Furthermore, the risk of hospital death based on the slope of the LAC_Δ24 _was greater in the increasing blood lactate concentration group (*n *= 1,879) and the mildly decreasing blood lactate concentration group (slope 0 to -1 mmom.L^-1^.24 hours^-1^, *n *= 1,680) when compared to a significantly decreasing blood lactate concentration (less than -1 mmom.L^-1^.24 hours^-1^, *n *= 1,482) (OR 1.66 (1.35 to 2.04), OR 1.29 (1.02 to 1.62) respectively).

**Table 3:** Multivariate models for the prediction of hospital and ICU mortality

Effect	Hospital mortality	*P*-value	ICU mortality	p-value
	Odds ratio (95% CI)		Odds ratio (95% CI)	
APACHE II score	1.13 (1.11 to 1.14)	< 0.0001	1.13 (1.11 to 1.15)	< 0.0001
Age (yr)	1.02 (1.02 to 1.03)	< 0.0001	1.01 (1.00 to 1.02)	0.004
LAC_TW24 _(mmom.L^-1^)	1.37 (1.29 to 1.45)	< 0.0001	1.43 (1.35 to 1.52)	< 0.0001
LAC_Δ24 _(mmom.L^-1^)	1.15 (1.10 to 1.20)	< 0.0001	1.18 (1.13 to 1.24)	< 0.0001
Diagnosis*		< 0.0001		< 0.0001
Mechanical ventilation	1.93 (1.52 to 2.45)	< 0.0001	2.81 (1.95 to 4.05)	< 0.0001
Hospital*		0.0001		0.006
Admission date (decreased risk per year)	0.91 (0.83 to 0.99)	0.04	0.88 (0.78 to 1.00)	0.05
Surgical patients	0.67 (0.53 to 0.84)	0.0007	0.80 (0.59 to 1.08)	0.15
Number of measurements	0.98 (0.95 to 1.01)	0.23	1.01 (0.97 to 1.04)	0.76
Female v male	0.96 (0.81 to 1.14)	0.66	0.99 (0.79 to 1.23)	0.90

*categorical variable, odds ratios excluded

APACHE II, Acute Physiology and Chronic Health Evaluation; LAC_TW24_, time weighted lactate; LAC_Δ24_, delta lactate-change in lactate in the first 24 hours

**Figure 3:**
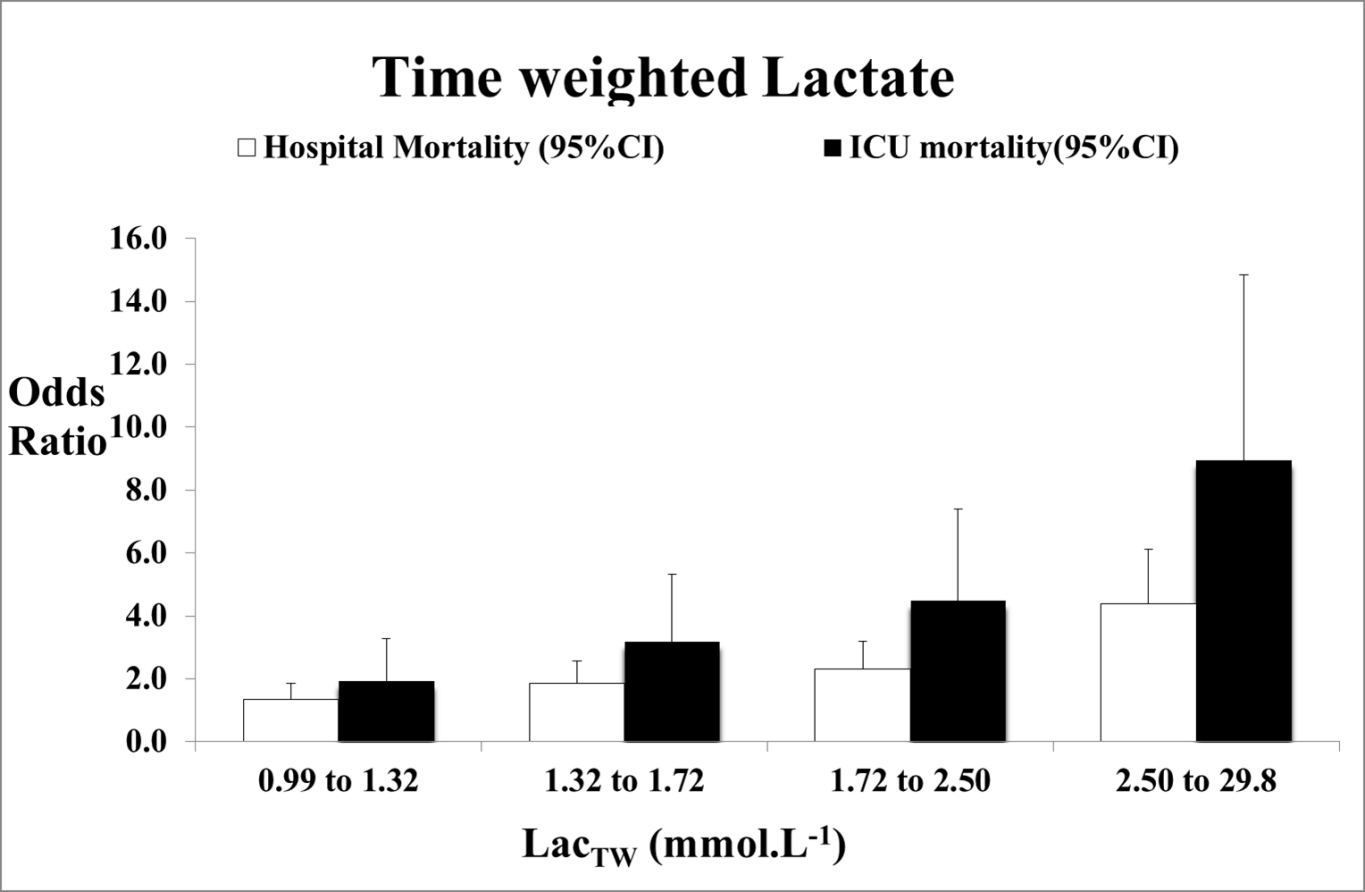
Adjusted* odds ratios for Lac_TW_referenced against the lowest quintile. * adjusted for APACHE II, diagnosis, age, gender, ventilation, surgery, admission date, hospital number of measurements. 95% CI, 95% confidence interval; APACHE II, Acute Physiology and Chronic Health Evaluation II; Lac_TW_, time weighted lactate over the first 24 hours.

For every one unit increase in LAC_TW24 _the risk of hospital death increased by 37% (OR 1.37 (1.29 to 1.45) *P *< 0.0001) and the risk of ICU mortality increased by 43% (OR 1.43 (1.35 to 1.52) *P *< 0.0001). When LAC_TW24 _was divided into quintiles and analysed as a categorical variable, each quintile progressively showed an increased risk in comparison to the lowest quintile for both hospital and ICU mortality (Figure 3).

When prediction equations excluding measurements of lactate were derived from 50% of the data and then applied to the remaining holdout sample, the AUC for hospital and ICU mortality was 0.820 ± 0.01 and 0.873 ± 0.01 respectively. The inclusion of static and dynamic measures of lactate homeostasis within the first 24 hours improved the prediction of hospital and ICU mortality (Table 4). When LAC_TW24 _and LAC_Δ24 _were added to the models of hospital mortality, the AUC-ROC was found to increase to 0.831 ± 0.01 and 0.825 ± 0.009 respectively (Table 4). In both cases, this represented a statistical (*P *< 0.0001) improvement in the prediction of mortality.

**Table 4:** Change in adjusted Area under the Receiver Operating Characteristic Curve

NAME	Hospital AUC_ADJ_	*P*-value*	ICU AUC_ADJ_	*P*-value*
(1) Established risk factors	0.820 ± 0.01		0.873 ± 0.01	
(1) + LAC_ADM_	0.822 ± 0.01	0.16	0.875 ± 0.01	0.24
(1) + LAC_Δ24%_	0.824 ± 0.01	0.006	0.879 ± 0.01	0.009
(1) + LAC_Δ24_	0.825 ± 0.01	0.005	0.879 ± 0.01	0.03
(1) + LAC_MAX24_	0.827 ± 0.01	0.007	0.883 ± 0.01	0.015
(1) + LAC_TW24_	0.831 ± 0.01	0.0001	0.887 ± 0.01	0.0005
(1) + LAC_MIN24_	0.832 ± 0.01	< 0.0001	0.886 ± 0.01	< 0.0001
				
(1) + LAC_TW _& LAC_Δ24 _	0.838 ± 0.01**	< 0.0001	0.895 ± 0.01**	< 0.0001

*compared to model (1) with established risk factors (APACHE II, diagnosis, age, gender, ventilation, surgery, date of admission and hospital)

**P < 0.01 compared to all individual measure of lactate

APACHE II, Acute Physiology and Chronic Health Evaluation II; Apache II, diagnosis, age, gender, ventilation, surgery, date of admission, mortality adjusting for established predictors of mortality; AUC_ADJ_, adjusted Area under the Receiver Operating Characteristic Curve; HVHF, high-volume hemofiltration; LAC_ADM_, admission lactate; LAC_TW24_, time weighted lactate; LAC_MAX24_, maximum lactate; LAC_MIN24_, minimum lactate; LAC_Δ24_, delta lactate; LAC_%Δ24_, percentage change in lactate in the first 24 hours

Interestingly, when LAC_MIN _(a static measure) was added to the models of hospital mortality, the AUC was found to increase to 0.832 ± 0.01 (Table 4). However, when we combined LAC_TW24 _and LAC_Δ24 _we found that this resulted in the greatest improvement in the prediction of both hospital and ICU mortality (Table 4), compared to any other combination.

## Discussion

### Statement of key findings

In a large multi-centre, heterogeneous cohort of critically ill patients, we examined whether dynamic indices of blood lactate concentration over the first 24 hours of ICU admission were independently associated with increased risk of hospital mortality. We found that LAC_TW24 _and the LAC_Δ24 _were the indices of lactate homeostasis within the first 24 hours that were independently the most predictive of hospital mortality. A rising, compared to a falling, blood lactate concentration over the first 24 hours was associated with a significantly increased risk of mortality. Furthermore, for every one mmol L^-1 ^increase in LAC_TW24 _and in LAC_Δ24 _the risk of hospital death increased by 37% and 15%, respectively. When LAC_TW24 _and LAC_Δ24 _were separately added to outcome prediction models, the AUC-ROC was found to increase significantly achieving close to 90% discrimination for ICU mortality. Furthermore, the maximal increase in AUC-ROC was achieved by the combination of both these dynamic indices (LAC_TW24 _and LAC_Δ24_) achieving close to 90% discrimination for ICU mortality and close to 85% discrimination for hospital mortality

### Comparison with previous studies-static versus dynamic

**Figure 4:**
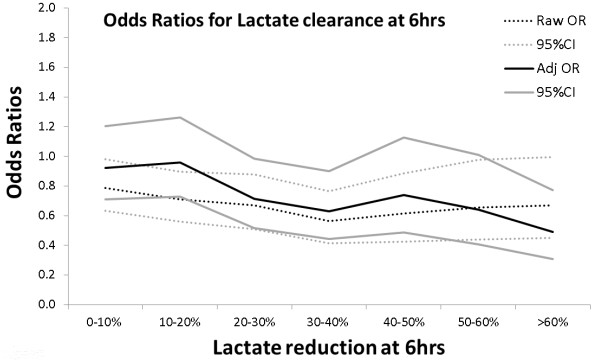
Raw and adjusted* odds ratios (95% CI) for death with Lac_Δ6 _referenced against zero change. * adjusted for APACHE II, diagnosis, age, gender, ventilation, surgery, admission date, hospital number of measurements. 95% CI, 95% confidence interval; APACHE II, Acute Physiology and Chronic Health Evaluation II; LAC_Δ24_, change in lactate in the first 24 hours; Lac_Δ6_, lactate clearance at six hours.

While quite a number of studies, including our previous study [4], have demonstrated that higher "static" indices of lactate derangement during ICU stay (LAC_TW24_, LAC_MAX24 _and LAC_MIN24_) are associated with higher ICU and hospital mortality [1,3,4,8,12] only a few studies have reported dynamic indices of lactate homeostasis. In these studies, sustained hyperlactataemia (generally regarded as more than six hours) was associated with higher mortality and, as the duration of this hyperlactaemia increased, the mortality rate also increased [2,5,12,13,16,17]. However, it must be noted that the majority of these studies were small, single centre, conducted in a single/specific diagnostic grouping (that is, severe sepsis), commonly examined for only short time periods after admission to ICU (under 24 hours) and were assessed in patients who had only a small number of lactate samples taken in the first 24 hours. These methodological deficiencies have confounded previous attempts to investigate the independent predictive value of dynamic indices of lactate in a heterogeneous population of critically ill patients. Our findings, however, for the first time demonstrate that early dynamic indices of lactate hoemeostasis (LAC_TW24 _and the LAC_Δ24_) are independently associated with hospital mortality in a large heterogeneous cohort of patients. Our study expands this association between decreasing blood lactate concentrations and mortality to all patient groups and up to the first 24 hours in the critically ill. Thus, not only the magnitude and the duration of lactate derangement (LAC_TW24_) but also the rate of derangement in blood lactate concentration over the first 24 hours (LAC_Δ24_) help identify patients at higher risk of death. In this regard, our findings significantly expand previous work which suggested that dynamic changes in lactate concentration could be clinically useful markers of patients at high risk of death [1,2,5,6,24]. However, to facilitate comparison with these previous studies we conducted a separate analysis over the first six hours after ICU admission and demonstrated that lactate clearance was inversely associated with increased risk of death at both univariate (*P *= 0.0001) and multivariate levels (*P *= 0.007) (Figure 4). These findings suggest for the first time that LAC_Δ24 _may be a clinically useful index of lactate homeostasis in the critically ill. While univariate results suggest a threshold value of 30 to 40% clearance over the first six hours, there is no clear indication from the multivariate model as the risk of death continues to decrease as the lactate clearance increases. Furthermore, these findings are consistent with previous studies suggesting that targeted reduction of lactate may be a beneficial strategy in the critically ill [14,17]. We also examined lactate clearance at 6 to 12 hours, 12 to 18 hours and 18 to 24 hours, but it was not found to be statistically significant at either a univariate or multivariate level (data not shown). In addition, while the majority of the previous studies examined patients with sepsis/septic shock, our cohort was heterogeneous. While we determined those with sepsis had an increased risk of mortality OR 1.60 (1.06 to 2.41) *P *= 0.02, there was no significant interaction between sepsis and LAC_TW24 _(*P *= 0.92) or sepsis and LAC_Δ24 _(*P *= 0.10). This indicates that there was no evidence in this cohort to suggest that the relationship between lactate and mortality differed significantly between those with and without sepsis.

To our knowledge this is the first demonstration that dynamic measures of lactate can be used to significantly improve the prediction of mortality in a heterogeneous cohort of critically ill patients. Moreover, both LAC_TW24 _and LAC_Δ24 _were positively associated with length of stay in survivors suggesting that these indices are robust and identify patients not only at high risk of mortality but also those who are at high risk of significant morbidity (data not shown). While individual dynamic measures do not outperform all the currently used static measures, the combined measure of lactate dynamics (LAC_TW24 _and LAC_Δ24_) could be the optimal early lactate variable to predict mortality as it can out-perform currently used static measures. This improved predictive ability may rest in the ability of this dynamic composite to assess the absolute magnitude, duration and rate of reduction of lactate derangement. Further studies are now required to confirm the clinical utility of this composite dynamic measure.

### Implications for clinicians

There is much interest in finding biomarkers that can assist in the early identification of patients who are, or continue to be, at high risk of death [25]. Our findings suggest that dynamic changes in blood lactate concentration over the first 24 hours may prove useful as a widely available biomarker of increased risk of death. Interestingly, in support of this hypothesis, goal directed resuscitation in critically ill patients with septic shock improves blood lactate concentrations over its six hours of therapy [26]. Furthermore, a prospective study which targeted reductions in blood lactate concentration goals was equally effective as a strategy targeting central venous oxygen saturation [25,27]. While our findings are in broad agreement with this association between 'physiological' normalisation of lactate and improved survival in the critically ill, further study is required before our findings should be used as a therapeutic target in mixed cohorts of patients and over varying time periods. However, clinicians confronted with a persistently abnormal blood lactate should also be aware of the fact that the absolute degree of derangement (duration and magnitude) and the rate increase of this derangement are associated with risk of death and should continue to maintain a high index of suspicion until these parameters have completely normalised.

### Limitations of the study

Our study is retrospective in design and thus potentially subject to systematic error and bias. However, all the clinical and electronic data utilised were collected prospectively in a large number of consecutive critically ill patients in four ICUs, are numerical in nature and were measured independently; thus, they were not amenable to selection bias or unintended manipulation. Furthermore, 71% of all ICU patients admitted during the study period (*n *= 7,155) had at least two blood lactate measurements (*n *= 5,041) and were included in our analysis, suggesting that our findings are generalisable and can be applied to the majority of ICU patients. A number of common therapeutic interventions, such as epinephrine [28], metformin [29], nucleoside analogues in HIV [30], high-volume hemofiltration (HVHF) with lactate-buffered replacement fluids [31], can all affect lactate levels and we did not have information on their use. However, the influence of these potential confounding factors is likely to be negligible given the small numbers of patients in which these factors would have been present, in relation to the size of the cohort; although presence of these factors might alter the applicability of these results to individual patients.

While our calculation of LAC_Δ24 _assumes that the kinetics of lactate is linear in nature, it is possible that it may follow an alternative decay profile, that is, exponential. However, we feel this pragmatic assumption does not positively bias the strength of the associations demonstrated. Furthermore, we believe that more complex modelling of lactate kinetics would obscure the take-home message for clinicians at the bedside of the association between rate of change of lactate concentration and mortality

The prognostic relevance of higher LAC_TW24 _and LAC_Δ24 _derives from the statistical examination of a large group of critically ill patients. Our results show that the inclusion of these variables can significantly improve the strength of predictive models of hospital mortality. This suggests that future investigators developing early prognostic models in the critically ill should consider the inclusion of these parameters (and the composite measure of both together). However, it must be noted that they should not be misused as a reliable prognostic sign in the individual patient, but in comparing groups of patients. In individual patients, higher LAC_TW24 _and Lac_Δ24_, however, should be considered a useful indicator pointing to the severity of illness and to superimposed complications.

### Future research

These dynamic lactate findings are novel and need to be confirmed by similar studies in other countries and large heterogeneous patient populations before they can be considered to reflect a general biological principle. Ideally, these studies should be conducted prospectively with simultaneous collection of information on interventions which may affect lactate. Furthermore, given the lactate indices assessed in this study are not necessarily the first in hospital lactate measurements and may not, therefore, consider the effects of early resuscitative interventions, future prospective studies should collect all lactate measurements (that is, including the emergency department) to determine if the demonstrated relationships are also observed during this earlier period.

In addition, if these studies confirm the value of LAC_Δ24_, then future studies could potentially focus on this marker as a surrogate endpoint to assess the success of early interventions in critically ill patients in the resuscitative period.

Future studies developing new or refining current severity of illness models in the critically ill should consider the inclusion of measures of lactate homeostasis in the first 24 hours. Our results suggest that the use of dynamic measures may result in the greatest improvement in predictive ability. In addition, it is worth noting that our study was potentially limited by having to add lactate measurements to the composite APACHE II score, rather than its component parts, and that a completely re-derived risk prediction equation that includes one or more index of lactate may further increase the predictive ability over that shown in this study.

## Conclusions

In conclusion, higher LAC_TW24 _and LAC_Δ24 _blood lactate concentrations are associated with greater hospital mortality. In the first 24 hours following ICU admission, they have significant independent predictive value, improve the performance of illness severity score-based outcome predictions and are superior to simple static measures of lactate concentration. Future studies should be conducted to determine the clinical utility of the composite measure of lactate derangement (magnitude, duration and rate of correction) to predict mortality and to determine potential the utility of LAC_Δ24 _as a future therapeutic target/trigger. While these dynamic measures do not have a routine role in daily clinical practice, clinicians should be especially alert to all patients with a persistently deranged or rapidly rising blood lactate concentration.

## Key messages

• Static derangements in lactate homeostasis during ICU stay have become established as clinically useful markers of increased risk of mortality.

• Dynamic indices of lactate homeostasis, which describe not only magnitude but also duration and trend over time, may be even more useful in predicting outcome.

• We demonstrated that higher lactate averaged over 24 hours (LAC_TW24_) and an increasing trend in lactate concentration over 24 hours (LAC_Δ24_) is associated with greater hospital mortality.

• Furthermore, the combination of these markers out-performed all individual 'static' indices of lactate homeostasis in predicting outcome in the critically ill.

• Clinicians should be especially alert in all patients with a persistently deranged or rapidly rising blood lactate concentration

## Abbreviations

APACHE II: Acute Physiology and Chronic Health Evaluation II; ANZICS-CORE: Australian and New Zealand Intensive Care Society-Centre for Outcome and Resources Evaluation; AUC-ROC: Area under the Receiver Operating Characteristic Curve; HVHF: high-volume hemofiltration; LAC_ADM_: admission lactate; LAC_TW24_: time weighted lactate; LAC_MAX24_: maximum lactate; LAC_MIN24_: minimum lactate; LAC_Δ24_: delta lactate; LAC_%Δ24_: percentage change in lactatein the first 24 hours.

## Competing interests

The authors declare that they have no competing interests.

## Authors' contributions

AN, MB, ME and RB carried out the database searches, participated in the data collation and drafted the manuscript with CF, ES, MR, GH and DJC. AN, RB, VP, DJC and MB conceived of the study, and participated in its design and coordination and helped to draft the manuscript. All authors read and approved the final manuscript.
